# Consensus-Statement der Österreichischen Gesellschaften für Pneumologie und Rheumatologie zur Definition, Evaluation und Therapie von progredient fibrosierenden interstitiellen Lungenerkrankungen (pfILD)

**DOI:** 10.1007/s00508-021-01874-3

**Published:** 2021-04-22

**Authors:** David Lang, Florentine Moazedi-Fürst, Judith Sautner, Helmut Prosch, Sabin Handzhiev, Klaus Hackner, Ivan Tancevski, Holger Flick, Hubert Koller, Hans Peter Kiener, Christian Prior, Bernd Lamprecht

**Affiliations:** 1 Klinik für Lungenheilkunde, Kepler Universitätsklinikum Linz, Krankenhausstraße 9, Linz, Österreich; 2 Klinische Abteilung für Rheumatologie und Immunologie, Landeskrankenhaus Universitätsklinikum Graz, Graz, Österreich; 3 2. Medizinische Abteilung mit Rheumatologie, Landesklinikum Stockerau, Niederösterreichisches Zentrum für Rheumatologie, Stockerau, Österreich; 4 Universitätsklinik für Radiologie und Nuklearmedizin, Klinische Abteilung für Allgemeine Radiologie und Kinderradiologie, Medizinische Universität Wien am Allgemeinen Krankenhaus der Stadt Wien, Wien, Österreich; 5 Klinische Abteilung für Pneumologie, Universitätsklinikum Krems, Krems, Österreich; 6 Universitätsklinik für Innere Medizin II, Infektiologie, Rheumatologie und Pneumologie, Medizinische Universität Innsbruck, Innsbruck, Österreich; 7 Universitätsklinik für Innere Medizin, Klinische Abteilung für Pulmonologie, Landeskrankenhaus Universitätsklinikum Graz, Graz, Österreich; 8 Abteilung für Atemwegs- und Lungenkrankheiten, Wiener Gesundheitsverbund – Klinik Penzing, Wien, Österreich; 9 Universitätsklinik für Innere Medizin III, Klinische Abteilung für Rheumatologie, Medizinische Universität Wien am Allgemeinen Krankenhaus der Stadt Wien, Wien, Österreich; 10 Facharztordination, Heiliggeiststr. 1, Innsbruck, Österreich

**Keywords:** Idiopathische Lungenfibrose, Exogen-allergische Alveolitis, Antifibrotische Therapie, Sarkoidose, Computertomographie, Immunsuppression

## Abstract

Interstitielle Lungenerkrankungen (ILD) sind eine heterogene Gruppe von Pathologien, die zunehmend als relevanter Faktor pulmonaler Morbidität und Mortalität erkannt werden. Verschiedene ILD wie die idiopathische Lungenfibrose (IPF), aber auch durch Autoimmunprozesse oder durch exogene Noxen bedingte ILD können zu progredienter, meist irreversibler Lungenfibrose führen. Die antifibrotischen Substanzen Nintedanib und Pirfenidon können den Krankheitsverlauf bei IPF-Patienten günstig beeinflussen. Dagegen werden ILD, die auf entzündlichen Prozessen wie z. B. rheumatologischen Grunderkrankungen oder exogen-allergischer Alveolitis beruhen, bis dato klassischerweise immunsuppressiv therapiert. Immer wieder kommt es aber trotz einer solchen Behandlung zu progredienter Fibrosierung. Eine positive Wirkung antifibrotischer Medikation auf progredient fibrosierende (pf)ILD abseits der IPF konnte in rezenten Studien demonstriert werden, auch wenn der Stellenwert der Antifibrotika in solchen Situationen noch nicht vollständig geklärt ist. Dieses Consensus-Statement beruht auf einem virtuellen, multidisziplinären Expertenmeeting von Rheumatologen, Pneumologen und Radiologen und wurde durch die jeweiligen ILD-Arbeitskreise der Österreichischen Gesellschaften für Pneumologie sowie Rheumatologie (ÖGP und ÖGR) akkordiert. Ziel war es, den aktuellen Stand von klinischer Praxis und wissenschaftlicher Datenlage zu Definition, Evaluation und Therapie von pfILD darzustellen. Zusammenfassend sollen ILD-Patienten einer standardisierten Abklärung unterzogen, in einem multidisziplinären ILD-Board diskutiert und dementsprechend therapiert werden. Kern dieser Empfehlungen ist, auch Non-IPF-Patienten mit dokumentiert progredient fibrosierendem ILD-Verlauf antifibrotisch zu behandeln, insbesondere wenn Honigwabenzysten oder eine bereits ausgedehnte Erkrankung vorliegen. Patienten mit fibrotischer ILD, die auf Basis der ILD-Board-Empfehlung primär keiner oder ausschließlich einer immunsuppressiven Therapie unterzogen werden, sollten engmaschig hinsichtlich eines progredienten Verlaufes überwacht werden.

## Einleitung

Interstitielle Lungenerkrankungen (ILD) sind eine heterogene Gruppe an Pathologien, bei denen es durch Inflammation und/oder Fibrose des pulmonalen Interstitiums durch unterschiedliche Ursachen zu einer Einschränkung der physiologischen Lungenfunktion kommt [1, 2].

Idiopathische Lungenfibrose (IPF) stellt die klassische Form einer idiopathischen progredient fibrosierenden (pf)ILD dar. Die IPF betrifft v. a. Männer im höheren Lebensalter (> 60 Jahre) mit Raucheranamnese und führt bei fast allen Betroffenen zu rasch progredienter Lungenfunktionseinschränkung mit ungünstiger Prognose [3, 4]. In den letzten Jahren kamen mit Pirfenidon und Nintedanib 2 antifibrotische Wirkstoffe zur Zulassung, die den Verlauf dieser Erkrankung bremsen können [5–8]. Da beide Medikamente den kontinuierlichen Lungenfunktionsverlust aber nicht gänzlich aufhalten können, ist eine frühe Diagnose der IPF mit rascher Therapieeinleitung essenziell.

Schon lange ist bekannt, dass auch andere fibrosierende ILD, allen voran die chronische exogen-allergische Alveolitis (EAA), die Sarkoidose und ILD im Rahmen von verschiedenen Autoimmunerkrankungen trotz adäquater Diagnose und – zumeist immunsuppressiver – Therapie einen progredient fibrosierenden Verlauf mit einer ebenso unvorteilhaften Prognose aufweisen können [1, 9–12].

Klassisch hierfür ist die systemische Sklerose (SSc), die sich in 70–80 % in der Lunge manifestiert, was zu einer substanziellen Prognoseeinschränkung führt [12–14]. Für die SSc-ILD wurde in den Scleroderma Lung Studies I und II die Wirksamkeit von Cyclophosphamid und Mycophenolat-Mofetil (MMF) nachgewiesen, wobei sich MMF als nebenwirkungsärmer herausstellte [15, 16]. Andere Therapieoptionen in therapierefraktären Fällen umfassen auch Tocilizumab, Rituximab, autologe Stammzelltransplantation und Lungentransplantation [12, 13, 17–20]. Im Jahr 2019 konnte in der SENSCIS-Studie an 576 SSc-ILD-PatientInnen mit Nintedanib analog zur IPF eine Reduktion des Verlustes an forcierter Vitalkapazität (FVC) gezeigt werden, was zur Zulassung dieser Substanz durch die Europäische Arzneimittel-Agentur (EMA) bei SSc-ILD führte. Patienten, die Nintedanib und zusätzlich eine Begleitmedikation mit MMF erhielten, wiesen den geringsten FVC-Abfall auf [21]. Dies legt nahe, dass zumindest bei einem Teil der SSc-ILD-Patienten eine Kombination aus antifibrotischer und immunsuppressiver Therapie vorteilhaft sein könnte.

Bei pfILD verschiedener Genese abseits der IPF wurden zuletzt in der INBUILD-Studie zu Nintedanib ähnliche Ergebnisse gezeigt: Auch hier konnte der Abfall der FVC signifikant reduziert werden, unabhängig davon, ob der bekannt ungünstige Prognosefaktor eines „usual-interstitial pneumonia“(UIP)-artigen Musters in der hochauflösenden Computertomographie (HRCT) vorlag [22]. In dieser Studie waren 663 PatientInnen mit verschiedensten progredient fibrosierenden ILD eingeschlossen, davon jeweils ca. ein Viertel mit EAA und autoimmun assoziierten ILD, zum Teil mit bestehender immunsupprimierender Basistherapie. Über alle Subgruppen hinweg konnten erneut konsistente Effekte bezüglich der FVC gezeigt werden [23]. Dies führte 2020 zur EMA-Zulassung von Nintedanib für die Indikation pfILD.

Auch für die Substanz Pirfenidon wurden in Studien an Patienten mit unklassifizierbarer oder mit Grunderkrankungen assoziierter pfILD ähnliche Ergebnisse berichtet [24, 25]. Aufgrund des Studiendesigns wurden hier aber die primären Endpunkte nicht erreicht, weswegen bei der EMA bis dato auch keine Zulassung in diesen Indikationen beantragt wurde.

Innerhalb weniger Jahre hat sich also die Therapielandschaft bei fibrosierenden ILD unterschiedlicher Genese deutlich erweitert. Speziell die antifibrotische Therapie mit Nintedanib – bis vor Kurzem IPF-Patienten vorbehalten – hat deutlich an Gewicht gewonnen. Wichtig sind daher nun einerseits die Definition solcher Erkrankungen und andererseits die standardisierte Abklärung und Diagnosestellung sowie in Folge die richtige Auswahl der Therapieoptionen.

Dieses Consensus-Statement soll dazu dienen, Abklärung und Therapie von pfILD-Patienten auf eine gemeinsame, aktuelle und evidenzbasierte Basis zu stellen.

## Methoden

Dieses Dokument beruht auf den Diskussionen im Rahmen eines virtuellen Expertenmeetings am 05.10.2020, an dem alle Autoren aktiv teilgenommen haben. Auf Basis der Aufzeichnung wurde das Manuskript von DL und BL verfasst und in 2 Review-Runden von allen anderen Autoren bearbeitet und schlussendlich akzeptiert.

Das Expertenmeeting wurde von Boehringer Ingelheim RCV GmbH & Co KG (BI) in Form eines Advisory Board Meetings unterstützt. BI hatte weder Einfluss auf den Prozess der Recherche, des Verfassens des Manuskriptes und der Überarbeitung durch die Autoren noch auf den Inhalt dieses Consensus-Statements. BI wurde die Gelegenheit gegeben, das Manuskript auf medizinische und wissenschaftliche Richtigkeit in Bezug auf die erwähnte BI-Substanz zu überprüfen.

Dieses Dokument erhebt nicht den Anspruch auf Vollständigkeit wie etwa eine Leitlinie. Vielmehr soll hier eine gemeinsame Diskussion über ein vielschichtiges Thema, welches multidisziplinär angegangen werden muss, möglichst komplett abgebildet werden.

Wir möchten betonen, dass die Abklärung und Therapie einzelner Erkrankungen, wie z. B. der IPF, EAA, der SSc-ILD oder RA-ILD, prinzipiell nach Maßgabe der entsprechenden Leitlinien in Zusammenarbeit der jeweiligen Fachdisziplinen erfolgen sollen.

## Definition von pfILD

Die häufigsten Ursachen von fibrosierenden ILD sind IPF, chronische EAA, mit Autoimmunerkrankungen assoziierte ILD, unklassifizierbare ILD und Sarkoidose, die Prävalenz wird zusammen bei ca. 50–70/100.000 angesetzt [1]. Abseits der in den meisten Fällen progredienten IPF wird davon ausgegangen, dass es bei anderen fibrosierenden ILD in bis zu 30 % zu einem progredienten Verlauf kommt [1, 9, 11].

In mehreren Übersichtsarbeiten und internationalen Positionspapieren wurde zuletzt klar empfohlen, pfILD abseits der IPF als „progredient trotz optimaler aktueller Abklärung und Therapie“ zu definieren [1, 11]. Dies bedeutet, dass der Terminus pfILD keine Diagnose darstellen soll, sondern lediglich einen Phänotyp beschreibt, der bei verschiedenen ILD auftreten kann. Jedenfalls sind weiterhin eine genaue Abklärung zur exakten Diagnosestellung und eine genaue Ursachenforschung insbesondere im Hinblick auf die EAA und auf Autoimmunerkrankungen durchzuführen.

**Figure 1:**
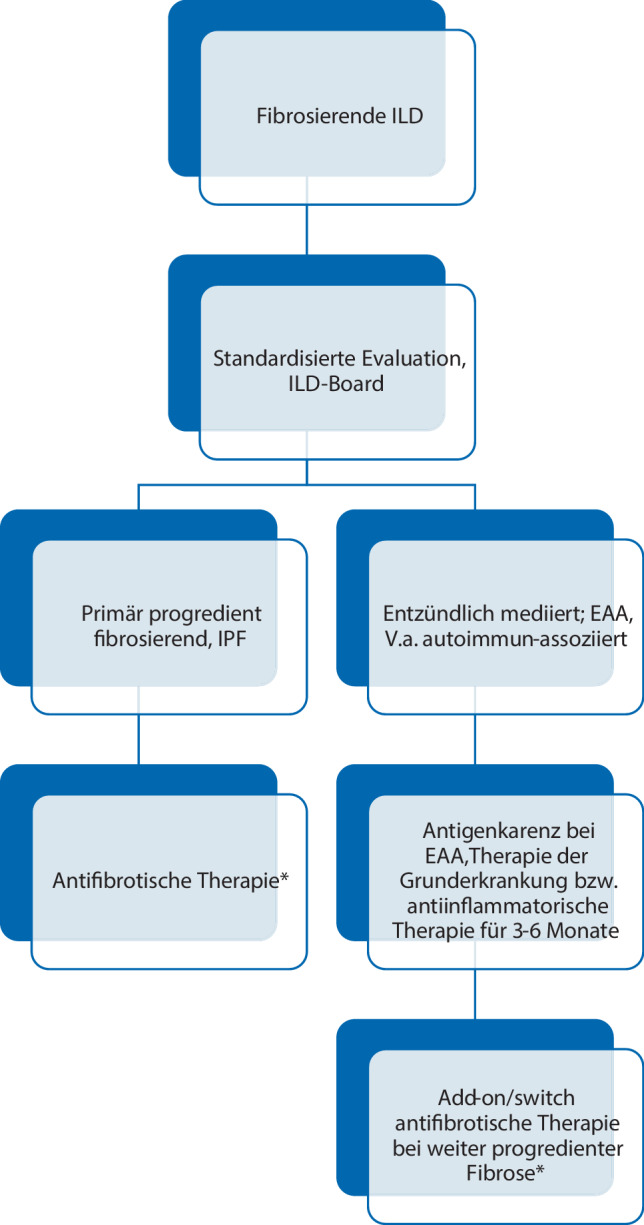

Wenn sich jedoch trotz dieser optimalen Aufarbeitung und ggf. trotz korrekt durchgeführter Therapie das Bild einer pfILD zeigt, besteht derzeit auf Basis der vorliegenden Studiendaten die Empfehlung zur Einleitung einer antifibrotischen Therapie. Eine EMA-Zulassung in dieser Indikation liegt auf Basis der INBUILD-Studie derzeit nur für Nintedanib vor [22] (Abb. 1).

## Standardisierte ILD-Abklärung

### Anamnese, Status

Die allermeisten pfILD-Patienten präsentieren sich mit progredienter Belastungsdyspnoe und Reizhusten. Symptome, die auf autoimmun-assoziierte Grunderkrankungen hinweisen können, wie Gelenkbeschwerden, Raynaud-Phänomen, Muskelschwäche oder Hauterscheinungen sollten gezielt abgefragt werden [1, 3]. Im Status ist die Auskultation essenziell, da die klassische „Sklerosiphonie“, also das spät-inspiratorische feine Knisterrasseln, sehr prädiktiv für das Vorliegen von Fibrose ist [1, 26].

In der Anamnese ist es wichtig, den Beschwerdebeginn zu erheben; so kann die Dynamik der Erkrankung abgeschätzt werden. Aktuelle und frühere Medikation sollte abgefragt werden (cave: insbesondere Amiodaron und Tumortherapien), ebenso Nikotinkonsum und sämtliche Expositionen im beruflichen und privaten Bereich, die auf eine EAA hinweisen können (organische Stäube z. B. im Stall, Vogelhaltung, Schimmel im Wohnbereich).

Ebenso soll eine Familienanamnese für pulmonale wie auch rheumatologische Erkrankungen erfolgen.

### Computertomographie

Die Diagnose einer ILD kann nur anhand einer hochauflösenden Computertomographie (HRCT) des Thorax gestellt werden [27, 28]. Die anatomische Verteilung der Veränderungen, die vorherrschenden Muster oder – wenn verfügbar – der zeitliche Verlauf können bereits wichtige Hinweise auf die zugrunde liegende Krankheit liefern [29].

Das klassische Thoraxröntgen eignet sich nicht zur spezifischen Diagnose einer ILD, kann aber helfen, den Verlauf der Erkrankung anhand von Vorbildern abzuschätzen oder Differenzialdiagnosen (z. B. kardiale Dekompensation) auszuschließen [27].

Hinweise auf eine fibrosierende Lungenerkrankung in der CT-Bildgebung sind netzartige (retikuläre) Veränderungen und/oder Milchglasverdichtungen. Beim zusätzlichen Nachweis von begleitenden peripheren Traktionsbronchiektasien/-bronchiolektasien ist eine Fibrose sehr wahrscheinlich. Als beweisendes Zeichen der Fibrose gelten Honigwabenzysten (Honeycombing) [27], die aber bei Weitem nicht bei jedem Patienten vorhanden sind, sondern je nach Entität nur in 30–40 % [30]. Die HRCT ermöglicht die Einteilung von ILD in verschiedene radiologische Muster, die ihrerseits für die Klassifizierung und Diagnose der ILD wegweisend sind. Häufige Muster sind die UIP, die durch peripher, subpleural und basal betonte Retikulation mit Honeycombing und Traktionsbronchiektasen gekennzeichnet sind und die nichtspezifische interstitielle Pneumonie (NSIP), die klassischerweise Milchglasverdichtungen und/oder retikuläre Verdichtungen in basaler und subpleuraler Verteilung mit begleitenden Bronchiektasien zeigt [27, 28].

**Figure 2:**
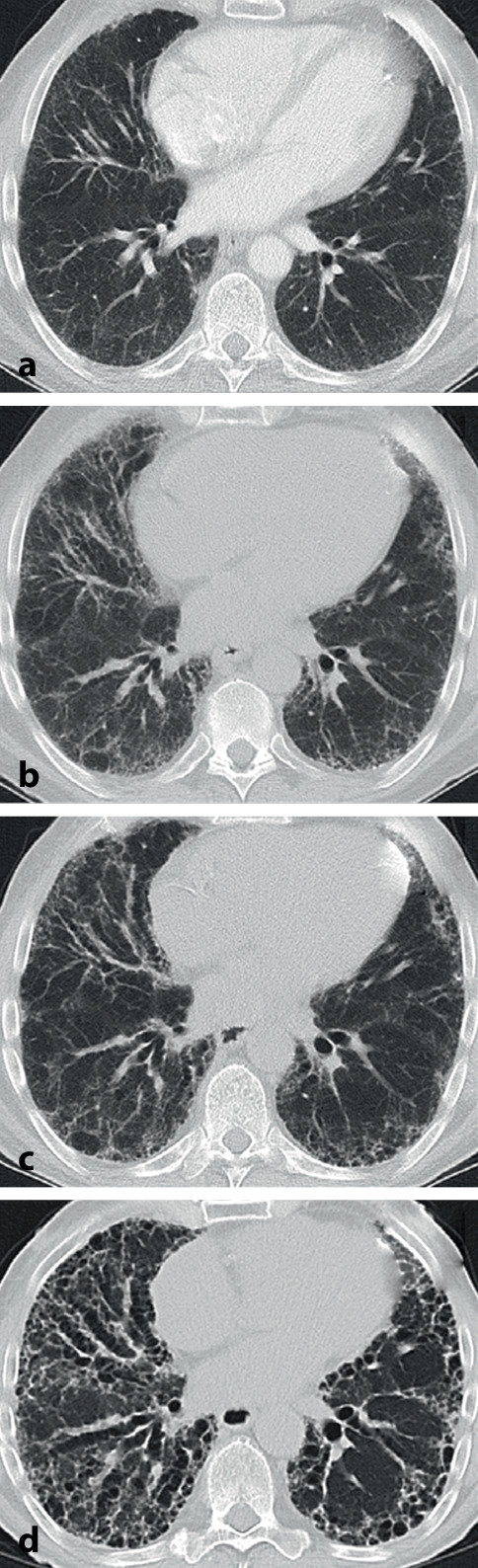

Wichtig ist aber, dass sich das radiologische Erscheinungsbild in der HRCT bei pfILD im Krankheitsverlauf verändern kann (Abb. 2) und dass auch schon bei Diagnosestellung Mischbilder verschiedener Muster vorliegen können. Die genaue Diagnose einer ILD benötigt also die Zusammenschau von HRCT mit klinischen Daten, wie z. B. einer genauen Anamnese hinsichtlich Antigenexposition, und anderen diagnostischen Tests wie Laboruntersuchungen und Lungenfunktionstestung.

**Figure 3:**
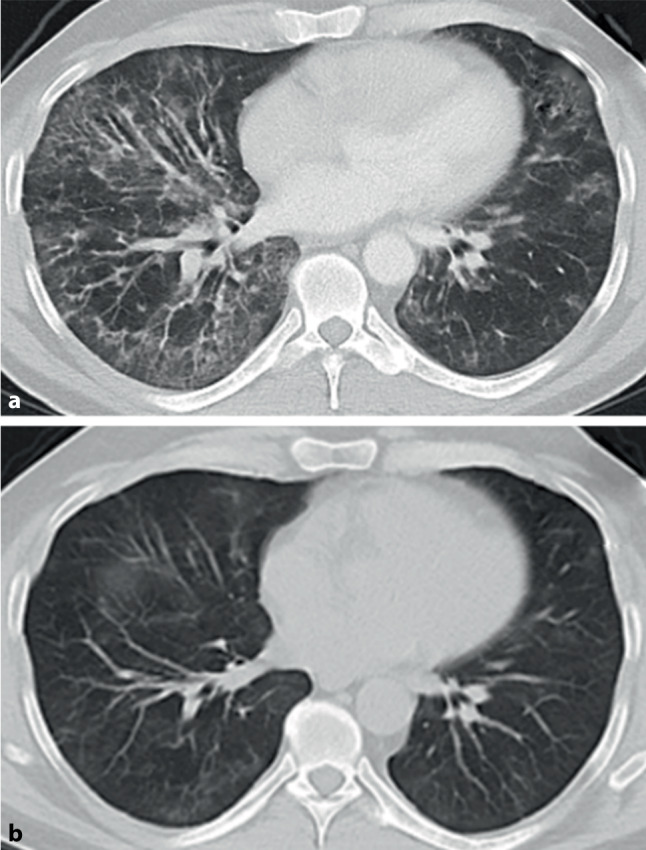

Neben der Unterscheidung verschiedener Muster ermöglicht die HRCT in gewissem Maße auch eine Abschätzung der Prognose [27]. Das klassische UIP-Muster mit Honeycombing und Traktionsbronchiektasen hat – ungeachtet seiner Ursache – die ungünstigste Prognose [1, 3, 27, 30]. Muster mit Milchglasverdichtungen oder retikulären Verdichtungen, wie z. B. das der NSIP, sind prognostisch günstiger und oftmals einer immunsuppressiven Therapie eher zugänglich (Abb. 3; [31, 32]) Dennoch können auch primär überwiegend inflammatorische Veränderungen später zu irreversibler Fibrose führen bzw. liegen oft von Beginn an Mischbilder aus fibrotischen und inflammatorischen Veränderungen vor.

Die HRCT kann zur Verlaufsbeurteilung bei pfILD herangezogen werden [27], allerdings sollten immer auch andere Verlaufsparameter, z. B. die Lungenfunktion einschließlich der Diffusionskapazität und die Symptomlast, berücksichtigt werden, da die Vergleichbarkeit von HRCT-Schnitten auch durch technische oder patientenseitige Faktoren eingeschränkt sein kann. Zukünftig könnten hier computerbasierte quantitative Auswertungsalgorithmen zum Einsatz kommen, die derzeit in der klinischen Routine aber noch nicht verfügbar sind [27, 33].

### Lungenfunktion

Jeder ILD-Patient soll eine umfassende Lungenfunktionstestung erhalten, um das Ausmaß der funktionellen Limitierung festzustellen und einen Ausgangsbefund für die weitere Verlaufskontrolle zu schaffen. Der Goldstandard hierfür ist die kombinierte Untersuchung von Spirometrie, Bodyplethysmographie und Diffusionskapazität, die Blutgasanalyse (BGA) sowie ein Leistungstest.

Wichtige Verlaufsparameter sind die FVC sowie als Ergänzung hierzu die Diffusionskapazität für Kohlenmonoxid (DLCO) [11, 34, 35]. Empfohlen wird auch die BGA in Ruhe und nach Belastung, was einerseits Rückschlüsse auf eine bestehende Sauerstoffpflichtigkeit (pO_2_ < 55 mm Hg bzw. < 60 mm Hg bei Zeichen der Rechtsherzbelastung) liefert und andererseits ein im Frühstadium oft noch okkultes Diffusionsproblem demaskieren kann [3, 36, 37]. In Kombination mit den Blutgasen sollte auch zumindest bei Diagnosestellung ein Belastungstest, wie z. B. ein 6‑min-Gehtest (6MWT), nach standardisiertem Protokoll durchgeführt werden [11, 38].

### Laborbefunde

Empfohlen ist bei ILD-Diagnosestellung eine Routinelabordiagnostik mit Blutbild, Leber- und Nierenfunktionsparametern. Daneben sollten Herz- und Muskelenzyme (Troponin, BNP, CK, CK-MB, Myoglobin, Aldolase und AST/ALT) im Hinblick auf kardiale Komorbiditäten und zur Evaluation einer möglichen Myositis abgenommen werden.

Eine serologische Routinetestung, die zumindest C‑reaktives Protein (CRP), antinukleäre Antikörper (ANA), antineutrophile zytoplasmatische Antikörper (ANCA), Rheumafaktor, Antikörper gegen citrullinierte Proteine (ACPA) und einen Myositisblot enthält, ist bei Verdacht auf IPF empfohlen und sollte auch bei Non-IPF-ILD evaluiert werden, insbesondere wenn der Verdacht auf eine autoimmunbedingte Genese besteht [1, 35, 39]. Bei klinischem Verdacht (z. B. SSc) oder auffälligen Routinebefunden sollte nach rheumatologischer Einschätzung eine zielgerichtete intensivere Testung des Autoantikörperprofils erfolgen.

Spezifische IgG-Antikörper gegen mögliche auslösende Antigene („Präzipitine“) können zur Erhärtung eines bestehenden klinischen und radiologischen Verdachtes auf EAA bestimmt werden [40].

### Bronchoskopie, BAL und Biopsie

Wenn durch die vorliegenden Befunde keine eindeutige Diagnose erhärtet werden kann, können invasive Maßnahmen zur Proben‑/Gewebegewinnung nötig werden.

Die bronchoalveoläre Lavage (BAL) ist insbesondere zur Abgrenzung entzündlich mediierter Prozesse sinnvoll und zeigt bei ILD mit Neigung zur Granulombildung wie bei Sarkoidose und bei aktiver EAA ein lymphozytäres Muster [41]. Ein hoher Neutrophilenanteil kann auf eine Infektion hinweisen, mäßig erhöhte Neutrophile und Eosinophile treten aber oft auch bei fibrotischem Bild wie bei IPF auf [35, 39].

Sollte eine Gewebeentnahme nötig sein, gibt es einerseits die Möglichkeit der bronchoskopischen transbronchialen Kryobiopsie und andererseits der chirurgischen Lungenbiopsie. Beide Verfahren weisen ein gewisses peri- und postinterventionelles Risiko auf und sollten daher nicht leichtfertig durchgeführt werden. Die Notwendigkeit einer invasiven Biopsieentnahme sollte im ILD-Board besprochen werden und eine genaue Abwägung von diagnostischem Nutzen und prozeduralem Risiko erfolgen [1, 35]. Das radiologische Muster in der HRCT und das histologische Muster müssen nicht immer konkordant sein; es können in Biopsien verschiedener Lungenpartien durchaus verschiedene histologische Muster wie NSIP und UIP nebeneinander vorkommen. Dies gestaltet insbesondere die histologische Klärung von fortgeschrittener ILD oft schwierig [11]. Jedenfalls soll eine Lungenbiopsie nur durchgeführt werden, wenn eine unmittelbare therapeutische Konsequenz besteht. In der klinischen Praxis ist dies v. a. in frühen ILD-Stadien gegeben, wo z. B. bei inflammatorisch mediierter ILD mit einer immunsuppressiven Therapie noch eine Verbesserung erzielt werden kann, oder bei EAA, wo dann die Antigenkarenz rasch etabliert werden könnte [11]. Die histologische Aufarbeitung sollte durch Pathologen mit großer ILD-Erfahrung an einem Referenzzentrum durchgeführt werden.

### ILD-Board

Abklärung und Diagnose von ILD sind nicht auf ein Fachgebiet zu beschränken, vielmehr bedarf dies einer multidisziplinären Zusammenarbeit [42]. ILD-Boards, zumeist bestehend aus Pneumologen, Rheumatologen, Radiologen und Pathologen, die der erforderlichen Multidisziplinarität Rechnung tragen sind an vielen Kliniken bereits Standard. Ihnen obliegt die diagnostische und therapeutische Entscheidung in den oftmals komplexen ILD-Fragestellungen [1, 11, 35, 39, 43]. Im Bereich der IPF konnte gezeigt werden, dass die Fallbesprechung im ILD-Board die diagnostische Sicherheit deutlich erhöht und auch prognostische Relevanz besitzt [35, 44, 45]. In den letzten Jahren sind auch ILD bei rheumatischer Grunderkrankung durch die zunehmenden therapeutischen Möglichkeiten und die steigende Awareness verstärkt in den Fokus gerückt. Umso wichtiger ist daher auch der Diskurs zwischen Pneumologie und Rheumatologie im ILD-Board geworden [1, 11, 13].

## Erfassung der Progredienz

Wichtig ist die Einholung von Vorbefunden, wie z. B. älteren Röntgenaufnahmen, CT-Bildern (z. B. auch Abdomen-CT mit Lungenanschnitten!) und Lungenfunktionsbefunden; so kann häufig ein bereits längerer, subklinischer Verlauf einer pfILD nachgewiesen werden.

Einige Risikofaktoren hinsichtlich Gefahr der Progression einer ILD können aus den Routinebefunden abgeleitet werden: So ist das UIP-Muster mit Honeycombing in der HRCT mit einer ungünstigeren Prognose verbunden [30], insbesondere bei Patienten mit RA-ILD oder EAA [46, 47]. Auch haben Patienten mit höherem Lebensalter generell eine schlechtere Prognose, ebenso wie Patienten mit bereits bei Beginn ausgedehnten fibrotischen CT-Veränderungen, eingeschränkter Lungenfunktion oder rascher Krankheitsprogression [11, 34, 48].

Es existiert noch keine klare Empfehlung, an welchen Biomarkern die Progression bei pfILD gemessen werden soll. Verschiedene, in klinischen Studien verwendete und vorgeschlagene Kriterien für pfILD sind in Tab. 1 aufgeführt.

**Table 1:** 

INBUILD-Studie (Nintedanib) [22]	pfILD außer IPF mit ≥ 10 % CT-Beteiligung	**In den letzten 24 Monaten trotz Standardbehandlung:**			
		Relat. FVC-Abfall ≥ 10 %	*Relat. FVC-Abfall ≥* *5* *% und:* Verschlechterung der Symptome oderProgress in CT		Verschlechterung Symptome und Progression in CT
Maher et al. (Pirfenidon) [24]	Unklassifizierbare pfILD	**In den letzten 6 Monaten trotz Therapie:**			
		≥ 5 % FVC-Verlust oder:	Symptomverschlechterung ohne andere Ursache (kardial etc.)		
RELIEF-Studie (Pirfenidon) [25]	pfILD inklusive CVD-assoz., fibrotischer NSIP, EAA, Asbest-induz. ILD	**Trotz adäquater Therapie:**			
		≥ 5 % FVC-Verlust/Jahr über zumindest 6–24 Monate in zumindest 3 Messungen			
George et al. („position paper“) [11]	Definition pfILD	**In den letzten 24 Monaten:**			
		FVC-Abfall ≥ 10 % relativ	*FVC-Abfall ≥* *5* *% relativ* *+*		Progrediente Symptome mit zunehmender Fibrose in CT
			DLCO-Abfall ≥ 15 %	Zunehmende Fibrose in CT oder zunehmende Symptome	

*pfILD* progredient fibrosierende ILD, *IPF* idiopathische Lungenfibrose, *ILD* interstitielle Lungenerkrankung, *CT* Computertomographie, *FVC* forcierte Vitalkapazität, *CVD* „collagen vascular disease“, *NSIP* nichtspezifische interstitielle Pneumonie, *EAA* exogen-allergische Alveolitis, *DLCO* Diffusionskapazität für Kohlenmonoxid

Für IPF wird die FVC derzeit als bester longitudinaler Parameter angesehen, da er mit dem Überleben korreliert und daher auch als primärer Endpunkt in den Studien bezüglich der Antifibrotika verwendet wurde [5, 6, 34]. Auch die DLCO korreliert mit der Mortalität bei IPF [34] und wird in der klinischen Routine wie auch in Studien herangezogen. Sie gilt als anfälliger für Schwankungen und Störungen, kann aber die FVC ergänzen [11]. Da alle gemessenen Lungenfunktionsparameter immer auch eine inhärente Schwankungsbreite besitzen, empfehlen wir, mehrere Funktionsparameter unterschiedlicher Messmethoden zu erheben, insbesondere auch Belastungstests wie den 6MWT [1, 11, 38]. Ebenso sollen die Beschwerdesymptomatik des Patienten und die subjektive Symptomwahrnehmung in die Entscheidungsfindung einfließen.

Unter erheblicher Diskussion steht derzeit das Zeitintervall, das abgewartet werden soll, bevor eine pfILD diagnostiziert werden kann: Es ist evident, dass pfILD-Patienten, die in die oben genannten Studien eingeschlossen wurden, in den Placebogruppen einen ähnlichen Lungenfunktionsverlust erlitten, wie IPF-Studienpatienten unter Placebo, obwohl sie im Durchschnitt jünger waren. Es erscheint daher sinnvoll, das Zeitintervall der Verlaufsbeobachtung vor Einleitung einer antifibrotischen Therapie möglichst kurz zu halten und bei Zeichen der Progression eher großzügig und rasch eine solche Therapie einzuleiten [9]. Ebenso unter Kritik stehen die relativ strengen Kriterien für Progression bei pfILD, welche in den Placeboarmen im gegebenen Zeitintervall oft gar nicht erreicht wurden [9]. Zu hinterfragen ist auch die starke Konzentration auf den Biomarker der FVC, insbesondere bei der beträchtlichen Fraktion an Patienten mit begleitendem Lungenemphysem, da dies die FVC gegenläufig verfälschen kann [9, 49, 50].

Es wurde daher vorgeschlagen, das Risiko der Progression nicht ausschließlich am vorherigen Verlauf festzumachen, sondern Hochrisikopopulationen schon anhand von initial vorhandenen Biomarkern zu identifizieren [9]. Deutliche Assoziationen mit der Mortalität bestehen hier schon bekannterweise für das Vorhandensein von Honeycombing [30] sowie ein Fibroseausmaß in der HRCT von > 20 % [9, 48, 51]. Bei Patienten mit diesen Befunden könnte die sofortige Einleitung einer antifibrotischen Therapie analog zur IPF bzw. ggf. auch eine Kombination mit immunsuppressiver Therapie bei autoimmun-assoziierter ILD erwogen werden, auch wenn (noch) keine Progression dokumentiert wurde [9]. Prospektive Daten zu einem solchen Vorgehen liegen jedoch derzeit noch nicht vor.

## Therapie und Management von pfILD

### Allgemeine Therapieziele

Therapieziel bei pfILD ist naturgemäß die Besserung von Symptomen, Lebensqualität und funktioneller Einschränkung, was aber nicht immer gelingen kann. Oftmals ist die Stabilisierung oder die Verlangsamung der Verschlechterung ein realistischeres Ziel. Da pfILD wie die IPF nicht heilbar sind und zumeist chronisch progredient verlaufen, ist immer – unabhängig von der medikamentösen Therapie und deren Erfolgsaussichten – auch schon parallel ein palliativer Therapieansatz zu verfolgen: Die individuell belastenden Symptome sollen priorisiert behandelt bzw. gelindert werden. Hierzu können verschiedenste Interventionen beitragen, von Rehabilitation zu psychologisch/psychotherapeutischer Betreuung bis hin zur Linderung von Atemnot mit Opiaten. Essenziell sind die enge Kommunikation mit Patienten und Angehörigen und die gemeinsame Erarbeitung der Ziele und Erwartungen. Bei allen medikamentösen Therapien muss der Nutzen auf den Krankheitsverlauf genauso wie die möglichen Nebenwirkungen und deren Auswirkungen auf die Lebensqualität besprochen werden [12].

### Medikamentöse Therapie

Die zielgerichtete Therapie von pfILD-Patienten sollte jedenfalls in einem ILD-Board besprochen werden. Hier sollten multidisziplinär sämtliche erhobenen Befunde aus der Abklärung durchgesehen werden und auch eine Einschätzung getroffen werden, ob sich die vorliegende ILD rein „fibrotisch“ im Sinne einer IPF verhält oder ob Zeichen einer aktiv inflammatorischen Erkrankung vorliegen.

Bezüglich der einzelnen Therapien in verschiedenen klinischen Situationen, z. B. ILD bei rheumatoider Arthritis, SSc oder EAA, sei auf die aktuellen Empfehlungen der relevanten Leitlinien verwiesen. Jede Therapie soll nach individueller Nutzen-Risiko-Abwägung, nach genauer Aufklärung und Diskussion mit Patienten und ggf. Angehörigen und in komplexen Fällen auch nach ILD-Board-Besprechung erfolgen.

Generell empfehlen wir, folgende Punkte in der Entscheidungsfindung bezüglich medikamentöser Therapie bei pfILD zu bedenken:

1. Nicht alle pfILD, abgesehen von IPF, benötigen eine sofortige Therapie. Bei langjährigem Verlauf, langsamer Progression und geringer Symptomlast, insbesondere bei älteren, multimorbiden Patienten, kann ein beobachtendes Procedere sinnvoller sein als ein nebenwirkungsreicher Therapieversuch.
2. Die diagnostischen Kriterien für die IPF und die daraus abgeleiteten Therapieindikationen bleiben aufrecht [35, 39, 52]. Im Hinblick auf die aktuelle Datenlage und die rezente Indikationserweiterung von Nintedanib auf pfILD [22] empfehlen wir, bei UIP-artigen Mustern, die nicht als IPF zu diagnostizieren sind, sehr engmaschig zu kontrollieren und bei Progredienz rasch eine antifibrotische Therapie einzuleiten. Dies betrifft insbesondere ILD-Patienten mit hohem Fibroseausmaß bei Diagnosestellung (> 20 % des Lungenvolumens) oder Vorhandensein von Honeycombing in der HRCT [9].
3. Bei Hinweisen auf eine zugrunde liegende Autoimmunerkrankung, soll jedenfalls ein/e Rheumatologe/in hinzugezogen werden und primär die Grunderkrankung bestmöglich therapiert werden. Zeigt sich im Verlauf dann dennoch ein progredient fibrosierendes Geschehen, ist eine antifibrotische Therapie als „add-on“ oder anstatt der spezifischen immunsuppressiven Therapie (je nach extrapulmonaler Krankheitsaktivität und Nebenwirkungsprofil) anzuraten.
4. Ähnliches gilt für Fälle von fibrosierender EAA: Hier ist das primäre Ziel die Identifikation der auslösenden Noxe und deren konsequente Vermeidung. Je mehr Hinweise auf aktuell inflammatorische Prozesse vorliegen (z. B. lymphozytäre BAL, Milchglas/Noduli in der CT), desto eher sollte auch eine immunsuppressive Therapie gegeben werden. Bei trotzdem anhaltend progredient fibrosierendem Verlauf sollte eine antifibrotische Therapie zusätzlich oder stattdessen angedacht werden.
5. Für alle anderen ILD, z. B. idiopathische NSIP oder unklassifizierbare ILD, gilt der obige Ansatz: Standardisierte Abklärung und Besprechung sowie Therapieentscheidung in einem ILD-Board; hier sollte dann nach oben genannten Gesichtspunkten abgewogen werden, ob:
  - eine medikamentöse Therapie sinnvoll ist,
  - primär ein Versuch mit immunsuppressiver Therapie erfolgen soll,
  - eine antifibrotische Therapie bei progredient fibrosierendem Phänotyp allein oder in Kombination mit (b) erfolgen soll.
6. Der Erfolg und die Verträglichkeit jeder eingeleiteten Therapie sollten spätestens nach 3 bis 6 Monaten reevaluiert werden. Gegebenenfalls kann dann eine Therapieadaptation, ein Wechsel bzw. ein „add-on“ erfolgen. Die Betreuung bzw. regelmäßige Vorstellung von pfILD-Patienten an einer spezialisierten ILD-Ambulanz ist vorteilhaft, da hier die üblicherweise langjährige Behandlungserfahrung vorhanden ist und ggf. Zugang zu klinischen Studien besteht.
7. Zu bedenken ist, dass die wissenschaftliche Evidenz für die Wirksamkeit immunsuppressiver Substanzen bei den meisten pfILD gering ist und solche Therapien ein oft deutliches Nebenwirkungsrisiko (z. B. Infektneigung, Verschlechterung von Komorbiditäten) mit sich bringen. Antifibrotische Therapien hingegen sind bei IPF (Pirfenidon, Nintedanib) [7, 8], SSc-ILD (Nintedanib) [21] und pfILD (Nintedanib) [22] mittlerweile in großen Studien erprobt und zugelassen. Die Rate und Intensität von Nebenwirkungen sind akzeptabel.
8. Kombinationen aus immunsuppressiver Therapie (insbesondere MMF, Methotrexat) und antifibrotischer Therapie (Nintedanib) erscheinen in der derzeitigen Datenlage als sicher [21, 22, 24]. Gewisse Subgruppen profitieren wahrscheinlich von einer solchen Kombination. Um mögliche Nebenwirkungen zu differenzieren, sollten diese Therapien aber nicht gleichzeitig, sondern sequenziell eingeleitet werden.

### Nichtmedikamentöse Therapien

Keinesfalls sollten neben diesen pharmakologischen Interventionen nichtmedikamentöse Maßnahmen vergessen werden. Diese umfassen insbesondere die Vermeidung des auslösenden Antigens bei EAA [1, 53, 54], Nikotinkarenz, regelmäßige ambulante oder stationäre Lungenrehabilitation [55] und Sauerstofftherapie, wenn indiziert. Eine Erstvorstellung an einem Lungentransplantationszentrum bei infrage kommenden Patienten muss zeitgerecht erfolgen. Ähnliches gilt für Patienten mit SSc, für die eine autologe Stammzelltransplantation in Betracht kommt. Sämtliche Impfungen laut Impfplan sollten aktuell durchgeführt bzw. ggf. aufgefrischt werden, insbesondere sind alle Patienten mit chronischen Lungenerkrankungen gegen Pneumokokken, Influenza, Pertussis und SARS-CoV‑2 zu impfen [56, 57].

## Interessenkonflikt

**D. Lang:** Vortragshonorare und Beratertätigkeit von/für BI, Bristol-Myers Squibb (BMS); Reise‑/Fortbildungsfinanzierung durch BI, Roche, Merck, Sharp & Dohme (MSD); **F. Moazedi-Fürst:** Vortragshonorare von BI, Pfizer, Roche, BMS, MSD, Lilly; **J. Sautner:** Beratertätigkeit von/für BI; **H. Prosch:** Vortragshonorare von BI, Roche, Novartis, MSD, BMS, AstraZeneca (AZ); Beratertätigkeit für BI, Roche, MSD, BMS, AZ; Reise‑/Fortbildungsfinanzierung von BI, Bayer; Forschungsfinanzierung durch BI; **S. Handzhiev:** Beratertätigkeit von/für BI und Roche; **K. Hackner:** Vortragshonorare von Novartis, AZ; Beratertätigkeit für Novartis; Reise‑/Fortbildungsfinanzierung durch Roche, BI; **I. Tancevski:** Vortragshonorare von BI, AZ, Novartis, Sanofi; Beratertätigkeit für BI, AZ, Novartis, Roche; Reise‑/Fortbildungsfinanzierung von BI, AZ, Novartis; **H. Flick:** Vortragshonorare von BI, Novartis, MSD, Roche, GlaxoSmithKline; Beratertätigkeit für BI, Reise‑/Fortbildungsfinanzierung von BI, Astropharma; **H. Koller:** Vortragshonorare von und Beratertätigkeit für BI; **H.P. Kiener:** Vortragshonorare und Beratertätigkeit von/für BIK: Vortragshonorare von BMS, Novartis, MedMedia Verlag, Mediaservice GmbH, MSD, BI; Beratertätigkeit für BI; **C. Prior:** Vortragshonorare von und Reise‑/Fortbildungsfinanzierung durch BI; **B. Lamprecht:** Vortragshonorare, Beratertätigkeit, Reise‑/Fortbildungsfinanzierung von/für BI und Roche.
